# Colonisation of antibiotic resistant bacteria in a cohort of HIV infected children in Ghana

**DOI:** 10.11604/pamj.2017.26.60.10981

**Published:** 2017-02-02

**Authors:** Eric Sampane-Donkor, Ebenezer Vincent Badoe, Jennifer Adoley Annan, Nicholas Nii-Trebi

**Affiliations:** 1 Department of Medical Microbiology, School of Biomedical and Allied Health Sciences University of Ghana, Accra, Ghana; 2 Department of Child Health, School of Medicine and Dentistry, University of Ghana Accra, Ghana; 3 Department of Medical Laboratory Science, School of Biomedical and Allied Health Sciences, University of Ghana, Accra, Ghana

**Keywords:** Antibiotic resistance, Ghana, HIV

## Abstract

Antibiotic use not only selects for resistance in pathogenic bacteria, but also in commensal flora of exposed individuals. Little is known epidemiologically about antibiotic resistance in relation to people with HIV infection in sub-Saharan Africa. This study investigated the carriage of antibiotic resistant bacteria among HIV infected children at a tertiary hospital in Ghana. One hundred and eighteen HIV positive children were recruited at the Korle-Bu Teaching Hospital in Ghana and nasopharyngeal specimens were collected from them. The specimens were cultured for bacteria, and the isolates were identified by standard microbiological methods. Antibiotic susceptibility tests were carried out on selected bacterial organisms by the Kirby Bauer method. Bacteria isolated from the study subjects included Moraxella catarrhalis (39.8%), coagulase negative staphylococci (33.1%), Streptococcus pneumoniae (30.5%), diptheroids (29.7%), viridian streptococci (27.1%), Staphylococcus aureus (22.0%), Citrobacter spp. (4.2%) and Neisseria meningitidis (0.9%). Prevalence of antibiotic resistance of S. pneumoniae ranged from 5.6% (ceftriaxone) to 58.3% (cotrimoxazole), M. catarrhalis ranged from 2.1% (gentamicin) to 80.6% (ampicillin), and S. aureus ranged from 7.7% (cefoxitin) to 100% (penicillin). The prevalence of multiple drug resistance was 16.7% for S. pneumoniae, 57.4% for M. catarrhalis and 84.6% for S. aureus. HIV infected children in the study area commonly carry multi-drug resistant isolates of several pathogenic bacteria such as S. aureus and S. pneumoniae. Infections arising in these patients that are caused by S. aureus and S. pneumoniae could be treated with ceftriaxone and cefoxitin respectively.

## Introduction

Antibiotic resistance is a global public health problem and is associated with increased hospitalizations, health costs and mortality [1–2]. The problem has been attributed to the misuse of antibiotics, which provide selective pressure favouring the emergence of resistant strains [1]. Antibiotic use not only selects for resistance in pathogenic bacteria, but also in the commensal flora of exposed individuals [3]. Antibiotic resistance of bacterial normal flora is important for two reasons. Firstly, antibiotic resistance genes can be transmitted from such commensal organisms to pathogens. Secondly, though infection with bacterial pathogens usually comes from an extrinsic source, they could also be from intrinsic sources where bacterial normal flora, which normally do not cause diseases become opportunistic causes of infections [4]. This is relatively common in people with immunosuppression such as HIV (Human Immunodeficiency Virus) patients. HIV infection has been reported to be a risk factor for colonization of antibiotic resistant organisms [5], though a lot more data is needed to clarify this. Majority of HIV infections occur in sub-Saharan Africa where there are high levels of antibiotic misuse. Little is known epidemiologically about antibiotic resistance in relation to people with HIV infection particularly, in the developing world. To gain some insights into antibiotic resistance in relation to HIV positive people in Ghana, this study was carried out. The aim of the study was to determine the bacterial flora colonising the nasopharynx of HIV positive children at a tertiary hospital in Ghana and the antibiogram of the organisms. It is expected that findings of the study would help contribute to effective antibiotic therapy of some of the bacterial infections arising in HIV positive children in the study area.

## Methods

This was a cross sectional study carried out at the paediatric HIV Clinic of the Korle-Bu Teaching Hospital (KBTH) located in Accra (capital city of Ghana) from February to April 2015. One hundred and eighteen HIV positive children less than 15 years were randomly recruited and nasopharyngeal swabs were collected from them. The nasopharyngeal specimens were cultured on Blood, Chocolate and MacConkey agars and the isolated organisms were identified based on colonial morphology, Gram stain and a battery of biochemical reactions [6]. Antibiotic susceptibility tests on the isolates were done by the Kirby Bauer method [7, 8], and the antibiotics tested included ampicillin, penicillin, erythromycin, cotrimoxazole, meropenem, chloramphenicol, cefuroxime, tetracycline, ciprofloxacin, ceftriaxone, cefoxitin, oxacillin and gentamicin (Oxoid Ltd., Basingstoke, UK). Briefly, the test isolate was emulsified in peptone until the turbidity was similar to that of 0.5% McFarland’s standard. A sterile cotton swab was dipped into the suspension and swabbed evenly across the whole surface of the agar plate in order to obtain a semi confluent growth. After incubation, the zones of inhibition around the antibiotic discs were measured and interpreted based on the breakpoint criteria of the Clinical and Laboratory Standards Institute [8]. Ethical approval for the study was obtained from the Ethical and Protocol Review Committee of the College of Health Sciences, University of Ghana and informed consent was provided by the parents of children participating in the study.

## Results

Demographic and clinical features of the hundred and eighteen (118) HIV positive children recruited in the study are reported in Table 1. The gender distributions of the study participants were similar and included 51.7% males and 48.3% females. Their mean age was 5.8 ± 3.3 years and majority of them were in the age group > 9-15 years (59.3%). Majority of them were Christians (83.9%) and attended school (86.4%). The mean CD4 counts of the study participants were 1088.9 cells/mm^3^ and 82.2% of them were on antiretroviral drugs. Culture of nasopharyngeal swabs collected from the study participants yielded several different organisms (Table 2). The most prevalent organism was Moraxella catarrhalis (39.8%), followed by coagulase negative staphylococci (33.1%), Streptoococcus pneumoniae (30.5%), diphtheroids (29.7%), viridian streptococci (27.1%) and Staphylocococcus aureus (22%). Antibiotic resistance testing was carried out on S. pneumoniae, S. aureus and M. catarrhalis. S. pneumoniae resistance decreased across cotrimoxazole (58.3%), tetracycline (33.3%), erythromycin (33.3%), oxacillin (27.8%), ceftriaxone (5.6%). M. catarrhalis resistance was highest for ampicillin (80.6%), followed by cotrimoxazole (60.0%), meropenem (42.6%), chloramphenicol (23.0%), cefuroxime (17%), tetracycline (17.0%), ciprofloxacin (14.9%), ceftriaxone (8.5%) and gentamicin (2.1%). S. aureus resistance was highest for penicillin (100%), followed by tetracycline (80.8%), cefuroxime (73.1%), erythromycin (38.5%), ciprofloxacin (19.2%), gentamicin (23.8%) and cefoxitin (7.7%). Four of the twenty-six S. aureus strains were methicillin resistant indicating a carriage of 3.4% of MRSA. The prevalence of multiple drug resistance (resistance to three or more classes of antibiotics) was 16.7% (9/36) for S. pneumoniae, 57.4% (27/47) for M. catarrhalis and 84.6% (22/26) for S. aureus.

**Table 1:** Demographic and clinical features of the study participants

Parameter	Number	%
Age (mean= 5.8±3.3 years)		
*<5 years*	21	17.8
*5-9 years*	27	22.9
*≥9 years*	70	59.3
Gender		
*Male*	61	51.7
*Female*	57	48.3
Current school attendance	102	86.4
Religion		
*Christian*	99	83.9
*Moslem*	19	16.1
Antiretroviral taken	97	82.2
Parameter	Number	%
Age (mean= 5.8±3.3 years)		
*<5 years*	21	17.8
*5-9 years*	27	22.9
*≥9 years*	70	59.3
Gender		
*Male*	61	51.7
*Female*	57	48.3
Current school attendance	102	86.4
Religion		
*Christian*	99	83.9
*Moslem*	19	16.1
Antiretroviral taken	97	82.2

Mean CD4 counts of study participants=1088.9 cells/mm^3^.

**Table 2:** Bacterial colonization of the nasopharynx of HIV infected children <15 years

Bacteria	N	%
*Moraxella catarrhalis*	47	39.8
Coagulase negative staphylococci	39	33.1
*Streptococcus pneumoniae*	36	30.5
Diptheroids	35	29.7
Viridan streptococci	32	27.1
*Staphylococcus aureus*	26	22
*Citrobacter spp.*	5	4.2
*Neisseria meningitidis*	1	0.85
		

N- number of subjects

## Discussion

In this study we investigated carriage of antibiotic resistant bacteria among HIV positive children at a tertiary hospital in Ghana. A widely diverse bacterial population was isolated from the HIV positive children and it may be worthwhile discussing the overall bacterial flora of the nasopharynx of this at-risk population. While M. catarrhalis was the commonest organism carried by the HIV positive children in this study (39.8%), in a similar study in Cambodia, M. catarrhalis was quite rare (6%) [9]. In the Cambodian study, S. aureus was the commonest organism isolated (30.4%), but in this study, it was the fourth common organism at a prevalence of 22%. These reflect variations in microbiota composition in different geographical regions and could be due to a wide range of factors. Members of the enterobacteriaceae were rarely isolated from the nasopharynx of the study participants, an observation that agrees with previous studies [9]. Like the Cambodian study [9], some of the common colonisers of the nasopharynx such as Neisseria meningitidis and Haemophilus influenzae were rarely isolated, which could be due to long-term routine vaccination against these organisms in many countries including Ghana. Antibiotic resistance has become a major public health problem especially with important pathogens such as S. pneumoniae and S. aureus.

Much of the recent interest in the epidemiology of pneumococci involves tracing the spread of penicillin resistance, as this has been the main drug of choice for treating pneumococcal infections for a long time. In this study, pneumococcal penicillin resistance was 33.3%, while previous studies in Ghana reported wide disparities of 0-66% [10–13]. The mechanism of resistance to penicillin and other beta-lactam antibiotics in pneumococci is attributed to variations in penicillin binding proteins and is disseminated among pneumococci through intraspecies or interspecies recombination, an event which is known to occur predominantly in the nasopharynx. It is important to note that ceftriaxone showed the lowest pneumococcal resistance, an observation, which concurs with previous studies [12, 13], and therefore highlights this antibiotic as a suitable choice for empirical treatment of pneumococcal infections in both healthy and HIV positive people. Pneumococcal resistance to cotrimoxazole was quite high (58.3%) but significantly lower than what had been previously reported in Ghana (100%) by several studies [13, 15]. This is interesting as cotrimoxazole is routinely administered in prophylaxis to HIV positive people [14].

This finding suggests that cotrimoxazole prophylaxis does not necessarily lead to antibiotic resistance of pneumococci carried by HIV positive people. Donkor et al. [15] have reported a similar observation about pneumococcal carriage and penicillin prophylaxis in relation to people with sickle cell disease. The extremely high percentage resistance (>80%) of S. aureus and M. catarrahalis to penicillin/ampicillin is due to the production of beta-lactamase by most strains of these bacteria that degrade the beta lactam ring of penicillin [16]. Methicillin resistant S. aureus is the most important S. aureus strain in terms of antibiotic resistance. In this study, MRSA carriage was 3.4%, which is significantly higher than the 0.3% carriage recently reported among healthy people in Ghana [17]. HIV infection has been identified as an independent risk factor for determining colonization with MRSA though the reasons for this are not clear. Despite the high levels of resistance to the several antibiotics tested, over 90% of the S. aureus isolates were susceptible to cefoxitin which indicates the suitability of this antibiotic for managing S. aureus infections among HIV positive children. Our data depicts carriage of highly resistant bacteria among the HIV positive children, which may be attributed to the fact that these patients are more likely to be hospitalised and also receive more courses of antibiotics, which are risk factors for antibiotic resistance. Generally, these findings have serious implications for antibiotic treatment of infections in HIV infected people in the study area, given their risk of infection with resistant bacteria. This is particularly problematic as antibiotic treatment options also tend to be limited in sub-Saharan Africa.

## Conclusion

The study concludes that bacteria that commonly colonised the nasopharynx of the HIV positive children were M. catarrhalis, coagulase negative staphylococci and S. pneumoniae. Antibiotic resistance of nasopharyngeal flora was high especially, for S. aureus. Ceftriaxone and cefoxitin may be suitable antibiotics for treating S. pneumoniae and S. aureus infections respectively in the HIV positive children. It is recommended that infections of the common organisms that colonised the study participants be monitored among HIV positive children in the study region. The main limitation of the study is that, for ethical reasons, a control group of non-HIV positive children could not be recruited in the study.

### What is known about this topic

- HIV infection is a risk factor for colonization of antibiotic resistant organisms but the available information pertains mainly to the developed world.

### What this study adds

- HIV infected children in Accra commonly carry multi-drug resistant isolates of several pathogenic bacteria;
- Ceftriaxone and cefoxitin may be suitable antibiotics for treating S. pneumoniae and S. aureus infections respectively in the HIV positive children in Accra.
